# Are Onconeural Antibodies a Clinical Phenomenology in Paraneoplastic Limbic Encephalitis?

**DOI:** 10.1155/2013/172986

**Published:** 2013-07-25

**Authors:** Hongliang Zhang, Chunkui Zhou, Limin Wu, Fengming Ni, Jie Zhu, Tao Jin

**Affiliations:** ^1^Department of Neurology, The First Bethune Hospital of Jilin University, Jilin University, Xinmin Street 71, Changchun 130021, China; ^2^Department of Neurobiology, Care Sciences and Society, Karolinska Institute, Novum, Plan 5, 141 86 Stockholm, Sweden; ^3^Department of Neurology, The Second Part of the First Hospital, Jilin University, Lequn Street, Changchun 130021, China; ^4^Neuroprotection Research Laboratory, Massachusetts General Hospital, Harvard Medical School, Charlestown, MA 02129, USA; ^5^Department of Radiotherapy, The First Bethune Hospital of Jilin University, Xinmin Street 71, Changchun 130021, China

## Abstract

Paraneoplastic neurological syndromes (PNSs) occur in patients with cancer and can cause clinical symptoms and signs of dysfunction of the nervous system that are not due to a local effect of the tumor or its metastases. Most of these clinical syndromes in adults are associated with lung cancer, especially small cell lung cancer (SCLC), lymphoma, and gynecological tumors. The finding of highly specific antibodies directed against onconeural antigens has revolutionized the diagnosis and promoted the understanding of these syndromes and led to the current hypothesis of an autoimmune pathophysiology. Accumulating data strongly suggested direct pathogenicity of these antibodies. The field of PNS has expanded rapidly in the past few years with the discovery of limbic encephalitis associated with glutamic acid decarboxylase (GAD) 65, the voltage (VGKC-gated potassium channel) complex, the methyl (N-NMDA-D-aspartate), alpha-amino-3-hydroxy-5-methyl-4-isoxazolepropionic acid (AMPA), and gamma aminobutyric acid (GABA) (B) receptors, and so forth. Despite this, the clinical spectrum of these diseases has not yet been fully investigated. The clinical importance of these conditions lies in their frequent response to immunotherapies and, less commonly, their association with distinctive tumors. This review provides an overview on the pathogenesis and diagnosis of PNS, with emphasis on the role of antibodies in limbic encephalitis.

## 1. An Overview of Paraneoplastic Neurological Syndromes

The idea that neural cells can be the target of autoimmune responses mediated by antibodies is still not well recognized in the medical community [1]. Paraneoplastic neurological syndromes (PNSs) are rare dysfunctions of the nervous system in patients with cancer, which are not due to a local effect of the tumor or its metastases. Most of these clinically defined syndromes in adults are associated with lung cancer, especially small cell lung cancer (SCLC), lymphoma, or gynecological tumors. Antibodies directed against onconeural antigens are frequently detected in patients with PNS. So far, these antibodies have been thought to be the only markers of the disease and not to play a role in the pathophysiology. However, the recent description of antibodies directed against membrane receptors or ion channels and playing a pathogenic role has challenged this concept. In case of antibodies targeting intracellular onconeural antigens, patients almost always harbor a tumor; some tumors might be found several years after the onset of neurological symptoms. However, it is not the case in the patients with antibodies targeting surface antigens (ion channels, receptors, or receptor associated proteins).

The reported incidence of PNS varies greatly since most estimates are from referral centers and not from population-based studies [2]. Paraneoplastic sensory neuropathy is probably the most common (3–7 per 1000 cancer diagnoses), followed closely by paraneoplastic encephalitis (3 per 1000) and cerebellar degeneration (2 per 1000) [3]. A rough classification of PNS is illustrated in Table 1 [4].

## 2. Limbic Encephalitis: An Increasingly Recognized Entity Belonging to PNS

The limbic system of brain comprises hippocampus, amygdala, hypothalamus, corpus mamillare, fornix, and gyrus cinguli (the Papez circuit) and is responsible for cognition, affect, and autonomic regulation. Limbic encephalitis was described for the first time by Brierley and colleagues in 1960 [5]. It is characterized by subacute onset (from days to several months) of short-term memory loss, disorientation, seizures, confusion, behavioral disturbance, psychiatric symptoms, and altered consciousness suggestive of involvement of the limbic system [6]. Less frequently, patients can have delusional thoughts and paranoid ideation [7], and some patients may have hyponatremia.

In the last decades, limbic encephalitis has been extensively investigated. According to the current knowledge, all types of limbic encephalitis fall into one of two main categories, infectious or autoimmune etiology. Infectious limbic encephalitis is caused by direct invasion of the brain by infectious agents, usually viruses, whereas autoimmune limbic encephalitis is caused by the individual's autoimmune reaction against itself. The current review will center on autoimmune limbic encephalitis and its clinical characteristics. Of note is that although the etiology was historically considered paraneoplastic, limbic encephalitis may also arise from nonparaneoplastic mechanisms, that is, autoimmune processes independent of malignancy. The clinical presentations are quite similar in the two groups. Prodromal flu-like symptoms may point to a nonparaneoplastic etiology, whereas smoking and weight loss suggest a paraneoplastic etiology [8]. The difficulty in differentiating the two categories stems from the fact that in 60% to 70% of paraneoplastic cases, neurological symptoms precede the detection of the tumor [9, 10].

**Figure 1:**
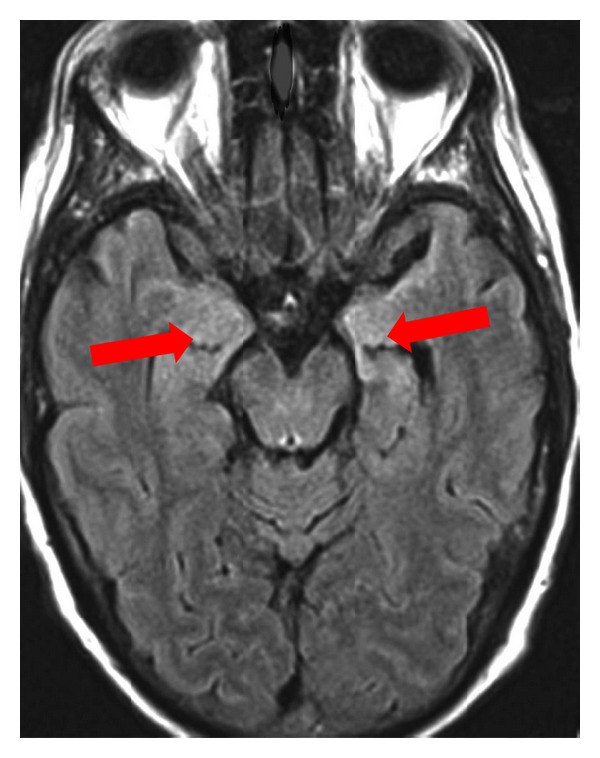
MRI FLAIR of a patient with limbic encephalitis and positive NMDAR antibodies in the CSF. Increased signal intensity is seen in the bilateral medial temporal lobes and hippocampi.

Established diagnosis of this syndrome after exclusion of infective and toxic disorders should prompt the initiation of immunotherapy [11]. The following investigations may aid an accurate diagnosis: analysis of cerebrospinal fluid (CSF), electroencephalogram (EEG), magnetic resonance imaging (MRI), positron emission tomography (PET), and detection of onconeural antibodies in the CSF and/or serum. CSF usually shows lymphocytic pleocytosis, increased protein concentration, and oligoclonal bands. Regardless of the type of clinical presentation, EEG is almost always abnormal, typically revealing focal or generalized slow wave abnormalities or epileptic form discharges in the temporal lobes [12]; T2-weighted or fluid-attenuated inversion recovery (FLAIR) MRI may show hyperintense signals of the medial temporal lobes, although other sites of lesions can also be detected (Figure 1); ^18^F-fluorodeoxyglucose (FDG) positron emission tomography (PET) may detect hypermetabolism in the medial temporal lobes, even when MRI is normal [12]; various antibodies may be present in serum and CSF. The information provided by the combination of clinical, EEG, MRI, and CSF routine studies suggests the diagnosis of limbic encephalitis in most patients with a classic presentation of the syndrome [12]. It is not mandatory that all investigations show pathological features, and not all cases of limbic encephalitis have typical MRI findings. However, if EEG, MRI, and CSF analyses are all normal, the diagnosis of limbic encephalitis is highly unlikely [8]. The diagnostic criteria for limbic encephalitis are listed in Table 2 [13], and the differential diagnoses of limbic encephalitis are summarized in Table 3.

Clinical characteristics of the different types of limbic encephalitis significantly vary according to the antibody type. NMDAR encephalitis often presents with cognitive and behavioral abnormalities. Because psychiatric symptoms are early and prominent, it is not rare for patients to be treated with antipsychotic drugs at onset. Subsequently, characteristic features develop, including movement disorders (orofacial dyskinesia, dystonia), seizures, speech disorder, autonomic dysfunction, central hypoventilation, catatonia, and depressed level of consciousness [8, 11]. Patients with PNS and LGI1-antibodies usually present with classic limbic encephalitis but may show some specific features, such as hyponatremia, rapid eye movement (REM), sleep behavioral disorders, or characteristic tonic seizures. Factually, the concept that limbic encephalitis is an inflammatory disorder strictly limited to anatomic regions of the limbic system is inaccurate. In this regard, these nonrestricted inflammatory boundaries are the rule rather than the exception, particularly when the limbic encephalitis is paraneoplastic. This is evidenced by many pathologic studies that have shown inflammatory infiltration distant from the limbic system. In these patients, a careful clinical evaluation almost always reveals signs of involvement of other areas of the nervous system that may remain mild or become more prominent than the symptoms of limbic dysfunction. For example, PNS in many patients with anti-Hu antibodies may start as limbic encephalitis that often evolves to encephalomyelitis with dorsal root ganglionitis.

## 3. Tumors That Are Associated with Limbic Encephalitis

In PNS, 50% to 80% of the patients present with neurological symptoms of PNS prior to diagnosis of tumors [15]. The associated tumors in PNS are a lung cancer in 50–60% of patients, usually SCLC (40–55%), and the associated tumor is a testicular germ cell tumor in 20% of patients. Other associated tumors include breast cancer, thymoma, Hodgkin's lymphoma, and teratomas [2]. In paraneoplastic limbic encephalitis, the most common tumors and corresponding antibodies are SCLC (anti-Hu, anti-CRMP5, and anti-amphiphysin), testicular cancer (anti-Ma2), thymoma (anti-CRMP5), and breast cancer (anti-amphiphysin) [16]. In men younger than the age of 50 years with anti-Ma2 antibodies, limbic encephalitis is almost always associated with testicular germ cell tumors, which however can be microscopic and difficult to detect.

As one of the classical PNS, limbic encephalitis can be diagnosed within less than 5 years before cancer is detected [14]. Removal of the tumor is critical for neurologic improvement or stabilization of symptoms in PNS. Therefore, tumor should be screened in patients with limbic encephalitis.

## 4. Antibodies Commonly Detected in Limbic Encephalitis

Tumor immunologists introduced the term “onconeural” antibodies to designate antibodies that target antigens present in neuroectodermal tissues and tumors [17]. These antibodies are unambiguously demonstrated by standardized tests, associated with limited subsets of tumors, and are present in several PNS types [1]. Since the 1980s, various onconeural antibodies have been discovered, which can serve as biomarkers for classic paraneoplastic syndromes [18]. Classical limbic encephalitides with temporal lobe seizures are associated with onconeural antibodies directed against the intracellular antigens. Onconeural antibodies are found in about 60% of the patients with paraneoplastic limbic encephalitis. The most frequent related antibodies are anti-Hu, anti-Ma2 (with or without Ma1), anti-amphiphysin, and anti-CRMP5. The majority of patients with anti-Hu antibodies have symptoms also suggestive of the dysfunction of areas of the nervous system outside the limbic system.

In recent years, the spectrum of chronic inflammatory brain diseases characterized by the presence of antigen-specific antibodies in serum and CSF has greatly expanded. Many patients with paraneoplastic limbic encephalitis previously characterized as “seronegative” have in fact antibodies against cell surface antigens. Recent studies indicated that most cases previously considered “seronegative” have, in fact, antibodies against surface antigens [19]. More and more cases such as glutamic acid decarboxylase (GAD) 65 antibody encephalitis [20], the voltage-gated potassium channel (VGKC) complex antibody encephalitis [21] (including LGI1 and Caspr2 antibodies), N-methyl-D-aspartate receptor (NMDAR) antibody encephalitis [22], alpha-amino-3-hydroxy-5-methyl-4-isoxazolepropionic acid receptor (AMPAR) [23], and gamma aminobutyric acid receptor GABA(B) antibody encephalitis [24] are recognized. In a few years, the number of onconeural antibodies described in PNS has increased dramatically. Antibodies to the components of VGKCs, NMDARs, AMPARs, GABA(B), mGluR5 receptor, and glycine receptors (GlyRs) can be identified in patients and are associated with various clinical presentations, such as limbic encephalitis and complex and diffuse encephalopathies [23, 25, 26]. These diseases can be associated with tumors, but some of them are nonparaneoplastic, and antibody assays can help with the diagnosis. The identification of these new antibodies (cell surface antigen associated) has allowed recognition of a syndrome with clinical and radiological features indistinguishable from “classic limbic encephalitis.” The course of the newly identified syndromes tends to be less severe and it is often possible to achieve complete recovery with prompt immunomodulatory treatment. The most representative condition is LGI1-encephalitis, previously known as limbic encephalitis with VGKC complex antibodies [27, 28].

These antibodies are directed against two categories of antigens: (1) intracellular antigens (Hu, Ma2, CRMP5, amphiphysin, etc.) and (2) cell surface antigens (the VGKC complex, NMDAR, AMPARs, GABABRs, mGluR5 receptor, GlyRs, etc.). Whereas the disorders related to the first category of antibodies are associated with cancer (lung, testis, etc.), prominent brain infiltrates of cytotoxic T cells, and limited response to treatment, the disorders related to the second category of antibodies are associated less frequently with cancer (thymoma, teratoma), seem to be antibody mediated, and respond significantly better to immunotherapy. These two antibodies have in common the association with idiopathic or paraneoplastic limbic encephalitis [23, 24]. Seven out of 15 (47%) patients with limbic encephalitis associated with GABA (B) receptor antibodies had an underlying tumor, usually an SCLC [24]. In limbic encephalitis associated with AMPAR antibodies, the frequency of cancer was 64%, with SCLC being the most common type, followed by thymoma and breast cancer [23]. These patients have a better prognosis than those with antibodies against intracellular proteins [29, 30].  Table 4 summarizes the common antibodies against onconeural antigens detected in PNS and their potentially associated tumors.

## 5. Do Antibodies Play a Pathogenic Role in Limbic Encephalitis?

A cancer-stimulated immune response that cross-reacts with neural tissue—onconeural immunity—is considered the principal pathologic mechanism for PNS [31]. Some cancer cells express proteins that are normally restricted to the nervous system. For example, when serum from a patient with limbic encephalitis was incubated with the patient's cancer cells and with a rat's brain tissue, antibody fixation to the same Ma proteins on both neurons and cancer cells could be observed [31]. Pathological examination of the nervous system showed loss of neurons in affected areas of the nervous system with inflammatory infiltration by CD4+ T helper cells and B cells in the perivascular spaces and cytotoxic CD8+ T cells in the interstitial spaces [32–34]. Examination of CSF frequently demonstrates pleocytosis, intrathecal synthesis of IgG, and oligoclonal bands, supporting an inflammatory or immune-mediated etiology.

The discovery of paraneoplastic antineuronal antibodies resulted in the general belief that these are immune-mediated disorders triggered by onconeural antigens expressed by tumor cells. Despite the clear clinical evidence that many of the syndromes described earlier are antibody mediated, there is lack of direct evidence showing that these antibodies are pathogenic in PNS. Support for a pathogenic role of antibodies comes from the fact that the target paraneoplastic antigens are expressed both in the tumors and in the affected regions of the nervous system. Furthermore, the size of tumors is usually small and they are heavily infiltrated with inflammatory cells. Interestingly, spontaneous remissions of carcinoma may occur at the time of neurological presentation [35, 36]. One study even found more limited disease distribution and better oncologic outcome in SCLC patients with paraneoplastic antibodies [37].

There are studies on the effects of the serum or CSF IgG antibodies on the neuronal function in cultured cells [22, 23, 38] or on brain slices, but the transfer of clinical or electrophysiological evidence of disease to experimental animals by either systemic or intrathecal injection has not yet been reported, with the exception of mGluR1-Ab in paraneoplastic cerebellar degeneration [39] and reports of GAD-65 or amphiphysin antibodies [40, 41]. In some PNSs, circumstantial evidence suggests that T-cell-mediated mechanisms play a major pathogenic role [42]. It has been suggested that the most important determinant of the underlying immunopathogenesis and responsiveness to immunosuppression is the antibody type and level of the affected individual, which may determine the response to treatment [1, 18, 43].

Specifically, striking differences have been found between disorders with antibodies against intracellular antigens versus those to neural surface antigens. Disorders with antibodies to intracellular antigens are considered poorly responsive to immunotherapy [18, 20] and may be mediated by cytotoxic T cells [18, 34]. On the other hand, disorders associated with antibodies against cell surface antigens, such as the VGKC-complex or NMDAR, often respond well to treatment [20, 44].

Some laboratory evidence supports the role of pathogenic B-cell responses in limbic encephalitis. NMDAR antibodies from patients have been shown to decrease the numbers of NMDAR in postsynaptic dendrites of cultured hippocampal neurons. One study suggested that anti-Hu antibodies induced apoptosis when applied to cultures of neuroblastoma or mesenteric cells [45]. There is also evidence, however, pointing to that paraneoplastic limbic encephalitis may be T-cell mediated, as Hu-specific T cells have been found in the blood and CSF [46], and there are cytotoxic infiltrates of T cells in the brain and tumor of the patients with anti-Hu antibodies-associated encephalomyelitis [47].

A pathogenic role could only be proven for those paraneoplastic antibodies that are directed against easily accessible antigens located on the cell surface. In these disorders, indirect lines of evidence support the view that the cellular immune responses against these antigens are responsible for the neurological damage [46, 48, 49]. The relative contribution of the cellular and humoral immunity to the clinical and pathological manifestations has not been displayed. The paraneoplastic antibodies may, in these cases, be surrogate markers for T-cell activation [50]. Elevated CD8/CD3 ratios in diseases were associated with antibodies to intracellular antigens and suggested a cytotoxic T-cell-driven pathomechanism. In diseases with antibodies to surface antigens, this finding supports a B-cell-related pathomechanism, with evidence of a complement-mediated pathogenesis in patients with VGKC-complex antibodies. Interestingly, this immunopathogenic dichotomy parallels other autoimmune disorders such as polymyositis and dermatomyositis, which have a predominant T-cell- and antibody-mediated pathogenesis, respectively [51]. These observations may contribute to a rational choice in immunotherapies for these disorders [52]. A totally different mechanism seems at work in paraneoplastic cerebellar degeneration in Hodgkin's lymphoma because the target antigens of the associated anti-Tr and anti-mGluR1 antibodies are not expressed in Hodgkin's tumor tissue [53]. Dysregulation of the immune response in Hodgkin's lymphoma and an etiologic role for viral infections have been postulated in this disorder [53].

Thus far, it is still unclear whether antibody-mediated PNS, for example, VGKC complex antibody-associated limbic encephalitis, is driven by serum or intrathecal antibodies. The absolute concentrations of antibodies against a certain onconeural antigen are usually higher in serum than in the CSF. Moreover, antibodies are not always detectable in the CSF. Ideally, both serum and CSF samples should be sent for antibody testing, but their relative utility in followup of patients is under debate. Intrathecal synthesis of IgG and oligoclonal bands can help pointing to an immune-mediated disorder before the results of specific antibodies can be obtained, but the oligoclonal bands are not always present at onset or even thereafter, and whether their presence is evidence for ongoing pathology or merely a secondary epiphenomenon is not yet clear. The intrathecal synthesis of antibodies can actually be assessed by the calculation of the amount of specific antibodies in the CSF relative to the total CSF IgG and by comparison with similar calculations in the serum. The ratio represents intrathecal synthesis and is often higher in some PNS. In favor of a role for systemic rather than intrathecal antibodies, animal experiments have shown that certain regions of the brain, that is, the hippocampus and the hypothalamus, seem to be particularly vulnerable, and it is notable that limbic encephalitis with VGKC-complex (LGI1 and Caspr2) antibodies and anti-NMDAR encephalitis usually start with symptoms originating from the temporal lobe cortex, even though the target antigens are present much more widely in the CNS. The former usually affects hippocampus, amygdala, and anterior temporal cortex, whereas the latter usually affects hippocampus, cerebral cortex, basal ganglia, and thalamus [54]. Until recently, only 50% of patients with limbic encephalitis and SCLC were found antibody positive, usually harboring anti-Hu antibodies or, less frequently, other onconeural antibodies [29].

Immunopathological analysis of various antibody-associated limbic encephalitis may help elucidate the underlying immunopathogenic mechanisms, whereas unfortunately there has been a lack of laboratory data [52]. Why is limbic encephalitis reversible in patients with NMDAR antibodies that are in frequent association with ovarian teratoma [44, 55]? Furthermore, how does one classify those patients with GAD-65 antibodies that are not paraneoplastic in origin, who suffer from limbic encephalitis or chronic temporal lobe epilepsy [20]?

An important issue is that a positive report for any well-characterized onconeural antibody has to be assessed according to the clinical setting. All these antibodies, particularly those associated with SCLC, can be found in the patients with cancer without PNS [56]. Therefore, one should still rule out other potential causes of the neurological syndrome that is being evaluated. Up to 16% of patients with SCLC without PNS have low titers of Hu antibodies, whereas in the patients with PNS and Hu antibodies, the titers are substantially higher [37]. 

## 6. Treatments of Limbic Encephalitis

The basic principles of paraneoplastic limbic encephalitis therapy are resection of the tumor or oncological treatment [10]. When a patient with tumor is found in association with a possible paraneoplastic disorder, removal of the tumor is critical for neurologic improvement or stabilization of symptoms. Antibodies against onconeural antigens are sensitive and should prompt an extensive tumor screening in antibody-positive patients. In the patients with limbic encephalitis associated with ion channel/receptor antibodies, immunosuppressive or immunomodulatory treatment is promising to improve the disease. Limbic encephalitides with antibodies against intracellular onconeural antigens do not normally respond to immunosuppressive treatment; only tumor therapy may stabilize the syndrome. Treatment of PNS still remains difficult. Anti-Hu-antibody-positive patients do not normally respond to immunosuppressive treatment. The only therapy that stabilizes these patients is perhaps the tumor treatment itself [16, 57]. In other PNSs associated with defined onconeural antibodies, only a few patients have beneficial effects after treatment [58]. Most patients with limbic encephalitis and ion channel receptor antibodies also respond to immunosuppressive or immunomodulatory treatment [22, 23, 27, 59]. Patients with limbic encephalitis and antibodies against cell surface antigens such as VGKC or NMDAR often respond to immunotherapies, such as corticosteroids, intravenous immunoglobulin (IVIG), or plasma exchange. Other therapy regimens that might be of relevance are rituximab, cyclophosphamide, and azathioprine [60]. In limbic encephalitis patients with intracellular antibodies, Ma2-positive patients may respond better to immunosuppression than patients with anti-Hu or anti-CRMP5 antibodies [61]. Immunotherapies for limbic encephalitis have been summarized in Table 5.

## 7. Some Conclusions and More Questions

This field of immune-mediated CNS diseases is exciting but also challenging. Ideally, antibody testing should be performed using internationally validated procedures so that the diagnosis can be made and treatments started as soon as possible in the hope of restoring health, limiting hospitalization, and optimizing outcomes. Systematic studies of the treatments are needed in order to establish the best practice. Experience with the recently described antibodies, with exception of those against NMDAR, is still relatively small. Therefore, their inclusion in one particular group of the proposed classification must be viewed with caution until more cases are described. Good clinical-immunological correlations are crucial to define the clinical syndrome that most likely associates with a particular antibody.

The so-far identified antibodies might only be the tip of the iceberg, with antibodies to other membrane ion channels or receptors awaiting recognition in future. Even now, the range of clinical features exhibited by patients with VGKC, NMDAR, aquaporin (AQP) 4, or GlyR antibodies is wide and includes most aspects of the nervous system. Researchers in this field must provide good clinical descriptions of the case series associated with the antibodies they study. This approach will help clinicians identify the clinical syndromes and make a rational decision on which antibodies to request. Moreover, more effective methods are required for the detection of onconeural antibodies [62].

## Figures and Tables

**Table 1:** Classification of paraneoplastic neurological syndromes.

Central nervous system	
Limbic encephalitis	
Encephalomyelitis	
Brainstem encephalitis	
Stiff-person syndrome	
Opsoclonus-myoclonus	
Subacute cerebellar degeneration	
Paraneoplastic visual syndromes	
Cancer-associated retinopathy	
Melanoma-associated retinopathy	
Paraneoplastic optic neuropathy	
Motor neuron syndromes	
Subacute motor neuronopathy	
Other motor neuron syndromes	

Peripheral nervous system	
Acute sensorimotor neuropathy	
Subacute sensory neuronopathy	
Chronic sensorimotor neuropathy	
Subacute autonomic neuropathy	
Paraneoplastic peripheral nerve vasculitis	

Neuromuscular junction and muscle	
Myasthenia gravis	
Lambert-Eaton syndrome	
Polymyositis/dermatomyositis	
Acute necrotizing myopathy	
Cachectic myopathy	
Neuromyotonia	

**Table 2:** Diagnostic criteria of paraneoplastic limbic encephalitis.

Criteria by Gultekin et al. [13]	
Pathological demonstration of limbic encephalitis, or all 4 of the following.	
(1) Symptoms of short-term memory loss, seizures, or psychiatric symptoms suggesting involvement of the limbic system	
(2) <4 yr between the onset of neurological symptoms and the cancer diagnosis	
(3) Exclusion of metastasis, infection, metabolic and nutritional deficits, stroke, and side-effects of therapy that may cause limbic encephalopathy	
(4) At least one of the following:	
(a) CSF with inflammatory findings	
(b) MRI FLAIR or T2 unilateral or bilateral temporal lobe hyperintensities	
(c) EEG with epileptic or slow activity focally involving the temporal lobes	

Criteria by the Paraneoplastic Neurological Syndrome Euronetwork [14]	

All 4 of the following items are met.	
(i) Subacute onset (days or up to 12 wk) of seizures, short-term memory loss, confusion, and psychiatric symptoms	
(ii) Neuropathologic or radiologic evidence (MRI, SPECT, PET) of involvement of the limbic system	
(iii) Exclusion of other possible etiologies of limbic dysfunction	
(iv) Demonstration of a cancer within 5 yr of the diagnosis of neurologic symptoms or the development of classic symptoms of limbic dysfunction in association with a well-characterized paraneoplastic antibody (Hu, Ma2, CRMP5, amphiphysin, Ri)	

**Table 3:** Differential diagnoses of limbic encephalitis.

Infectious disorders	
Herpes simplex virus encephalitis	
Neurosyphilis	
Progressive multifocal leukoencephalopathy	
Rabies	
Creutzfeldt-Jakob disease	

Metabolic disorders	
Metabolic encephalopathy (uremic, hepatic, Cushing syndrome, etc.)	
Wernicke-Korsakoff syndrome	
Hashimoto's encephalopathy	

Systemic autoimmune disorders	
Sjögren syndrome	
Systemic lupus erythematosus	
Antiphospholipid syndrome	

Malignancies	
Lymphoma	
Glioma	
Gliomatosis cerebri	

Degenerative disorders	
Alzheimer's disease	
Lewy-body dementia	
Frontotemporal dementia	

Others	
Stroke with posterior cerebral artery involvement	
Central nervous system vasculitis	
Temporal lobe epilepsy	
Nonconvulsive status epilepticus	
Transient global amnesia	
Acute demyelinating encephalomyelitis	
Posterior reversible encephalopathy syndrome	
Intoxication (alcohol, lithium, etc.)	
Alcohol withdrawal syndrome	
Psychiatric disorder	

**Table 4:** The common antibodies detected in PNS and their associated tumors.

Antibodies	PNS	Associated tumors

Antibodies against intracellular antigens		
Anti-Hu	sensory neuronopathy, LE, BSE, encephalomyelitis	SCLC
Anti-Yo	SCD	Gynecological cancer
Anti-Ri	Opsoclonus-myoclonus, BSE	Breast cancer
Anti-Ma2	BSE, LE	Testis cancer, SCLC, breast cancer
Anti-CRMP5	SCD, chorea, myelitis, LE, sensory neuronopathy, optic neuritis	SCLC, thymoma
Anti-amphiphysin	SPS, myelitis, SCD, sensory neuronopathy	SCLC, breast cancer
Anti-GAD-65	SPS, myelitis	SCLC, breast cancer
Anti-SOX-1	LEMS	SCLC

Antibodies against cell surface onconeural antigens		
Anti-VGCC	SPS, LEMS	SCLC
Anti-VGKC complex	LE	SCLC, thymoma
Anti-NMDA receptor	LE	Teratoma
Anti-AMPA receptor	LE	SCLC, breast cancer, thymoma
Anti-AQP-4	NMO spectrum disorders	SCLC, breast cancer, thymoma
Anti-GABA-B receptor	LE	SCLC
Anti-CAR	Retinopathy	SCLC, melanoma, gynecological cancer
Anti-contactin-associated protein 2	Morvan syndrome	Thymoma
Anti-AchR/MuSK/RyR/Titin	MG	Thymoma

AMPA: amino-3-hydroxyl-5-methyl-4-isoxazole-propionate; AQP-4: aquaporin 4; CAR: cancer-associated retinopathy; CRMP5: collapsin response mediator protein 5; GABA-B: gamma-aminobutyric acid B; GAD-65: glutamic acid decarboxylase 65; LE: limbic encephalitis; LEMS: Lambert-Eaton myasthenic syndrome; MG: myasthenia gravis; NMDA: N-methyl-D-aspartate; NMO: neuromyelitis optica; SCD: subacute cerebellar degeneration; SCLC: small cell lung cancer; SPS: stiff-person syndrome; VGCC: voltage-gated calcium channel; VGKC: voltage-gated potassium channel.

**Table 5:** Immunotherapies for limbic encephalitis.

Acute phase of the disease	
First-line therapies	
High-dose corticosteroids	
Intravenous immunoglobulins	
Plasma exchange	
Second-line therapies	
Rituximab	
Cyclophosphamide	

Maintenance therapy	
Steroids	
Azathioprine	
Mycophenolate	
